# Long-term Effects of an Evidence-based Guideline for Emergency Management of Pediatric Syncope

**DOI:** 10.1097/pq9.0000000000000361

**Published:** 2020-10-26

**Authors:** Kristen H. Shanahan, Michael C. Monuteaux, Dalton Brunson, Sabrina E. Guse, Mark E. Alexander, John J. Porter, Mark I. Neuman, Andrew M. Fine

**Affiliations:** From the * Division of Emergency Medicine, Boston Children’s Hospital, Harvard Medical School, Boston, Massachusetts; † Harvard University, Cambridge, Massachusetts; ‡ Division of Emergency Medicine, Children’s National Hospital, George Washington University Medical School, Washington, DC, and; § Department of Cardiology, Boston Children’s Hospital, Harvard Medical School, Boston, Massachusetts.

## Abstract

Variability exists in the management of childhood syncope as clinicians balance resource utilization with the need to identify serious diseases. Limited evidence exists regarding the long-term impact of evidence-based guidelines (EBGs) on clinical practices. This study’s objective was to measure long-term changes in the management of syncope after implementing a syncope EBG in a single pediatric emergency department following the redistribution of resources to facilitate compliance over time.

**Methods:**

We included healthy patients aged 8–22 years, presenting to the pediatric emergency department with syncope between 2009 and 2017. Interrupted time series analysis compared testing rates and length of stay among the pre-EBG, short-term follow-up, and long-term follow-up periods.

**Results:**

The study included 1,294 subjects. From the pre-EBG period to the long-term follow-up period, recommended electrocardiogram and urine pregnancy test rose significantly [level change odds ratio (95% confidence interval) 5.56 (1.73–17.91) and 3.15 (1.07–9.32), respectively]. Testing and management not recommended by the EBG decreased significantly, including complete blood count, electrolytes, point-of-care glucose, chest radiograph, and intravenous fluids [level change odds ratio (95% confidence interval) 0.19 (0.09–0.40), 0.15 (0.07–0.32), 0.38 (0.18–0.81), 0.17 (0.06–0.49), and 0.18 (0.08–0.39), respectively]. Length of stay declined significantly. No delayed diagnoses occurred.

**Conclusions:**

Sustained improvements in syncope management persisted during long-term follow-up of the EBG despite minimal resources. The EBG was associated with increased focused evaluation and decreased low yield testing. EBGs may be useful tools to influence sustained clinical practices to promote safe, cost-effective, and high-quality care.

## Abstract 2

Supplemental Digital Content is available in the text.

## INTRODUCTION

Syncope occurs in up to 40% of children.^1^ Extensive evidence supports that the majority of syncope is a typical neurally-mediated syncope. Despite the mostly benign nature of pediatric syncope,^2,3^ clinicians often obtain low yield, high cost, and resource-intensive diagnostic evaluations in these children.^4–7^ Rare, life-threatening etiologies of syncope, including cardiac disease, may drive clinicians to pursue unnecessary evaluation in the absence of concerning history or physical findings.^8^ Wide variation in clinical practice and resource utilization for pediatric syncope exists in the emergency department (ED).^9^ Therefore, the pediatric ED staff at Boston Children’s Hospital implemented an evidence-based guideline (EBG) for the evaluation and management of pediatric syncope in July 2011.^10^ The EBG for syncope is one of several EBGs associated with short-term changes in clinical practices.^11–13^ In the year following implementation of the syncope EBG, clinicians obtained significantly fewer evaluations not routinely recommended in the EBG and obtained significantly more of the recommended tests.^10^ These improvements in guideline adherence and clinical care occurred in the setting of resources dedicated to EBG implementation.

Limited data exist on the long-term influences of EBGs on clinical practices.^14^ The reduction of intensive EBG-dedicated resources may affect adherence to the guideline over time. Lack of evidence regarding the long-term effects of EBGs represents a critical gap in knowledge. This study’s objective was to characterize the long-term changes in the diagnostic evaluation and management of syncope in children in the pediatric ED following the implementation of an EBG. We hypothesized that in the absence of resources dedicated to the EBG, clinicians would exhibit a decrease in adherence to the EBG recommendations in the long-term follow-up period.

## METHODS

### Evidence-based Guideline for Management of Syncope in the Pediatric Emergency Department

**Fig. 1.**
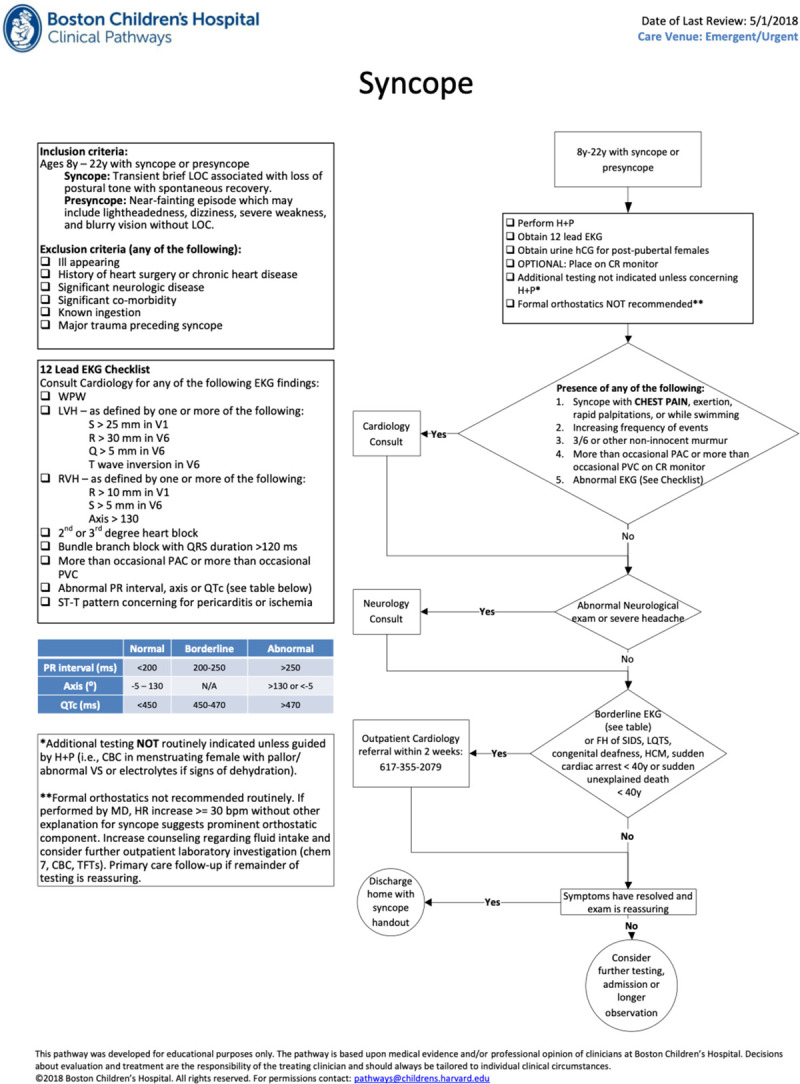
EBG for the management of syncope in the ED at Boston Children’s Hospital.

In July 2011, clinicians introduced an EBG for the evaluation and management of pediatric syncope in the pediatric ED at Boston Children’s Hospital, a large, urban tertiary care children’s hospital with an annual volume of approximately 60,000 visits per year. The guideline has been previously published (Fig. 1).^10^ Approximately 62 board-certified pediatric emergency medicine physicians, 14 board-certified general pediatricians, 18 pediatric emergency medicine fellows, and over 200 rotating residents from 4 pediatric and emergency medicine residency programs staff this pediatric ED. Other staff include rotating medical students, over 100 nurses, and 40 clinical assistants. The EBG routinely recommends an electrocardiogram (ECG) for all patients and urine pregnancy testing for post-menarchal females. The EBG does not routinely recommend serum laboratory testing, radiographic imaging, intravenous (IV) fluids, medications, or hospital admission. It provides focused recommendations for urgent or elective neurology or cardiology consultation.

A multi-disciplinary collaborative group that included clinicians from emergency medicine, cardiology, neurology, and nursing created the guideline by incorporating the best available evidence from the literature. A pediatric emergency physician and nurse led the iterative process, including 12 revisions over 1 year. Local experts from cardiology and neurology vetted and disseminated the guideline to inform their colleagues and consulting fellows of the collaborative effort optimally.

The introduction of the EBG included paper and electronic copies of the algorithm, and an associated order set in the electronic medical record. The authors of the guideline promoted the EBG at a staff meeting, sent e-mail reminders, and distributed a pocket-card to faculty and trainees at the time of implementation. At this time, a temporary multi-disciplinary team dedicated time and resources to EBG implementation.^10^

There were minimal long-term resources dedicated to promoting EBG adherence by clinicians working in the ED. Currently, the Division of Emergency Medicine has 45 guidelines, which have been recently termed “clinical pathways.” An attending physician serves as the champion for each clinical pathway. Champions complete a review of quarterly logs to assess for regressions to prior practices and review new relevant literature to inform an annual update if indicated. The champion circulates the annual updates to emergency medicine staff by email or presentation at a weekly departmental educational conference. New providers and trainees complete orientation videos, which includes a highlight of the departmental website containing all of the guidelines, and the associated order sets. The video does not specifically highlight the guideline and order set for syncope. During annual performance evaluations, the division chief provides attending physicians in emergency medicine with metrics on their adherence to selected clinical pathways. The division chief selects different pathways annually and does not inform the physicians of the selections ahead of time.

### Study Design

The Committee for Clinical Investigation at Boston Children’s Hospital approved this retrospective cross-sectional time-series study. The study included children aged 8–22 years who presented to the ED at Boston Children’s Hospital with syncope or pre-syncope from July 2009 to December 2017. The definition of syncope is a transient, brief loss of consciousness associated with loss of postural tone with spontaneous recovery.^15–17^ Pre-syncope is a near-fainting episode, which may include lightheadedness, dizziness, severe weakness, and blurry vision without loss of consciousness. The study included children with syncope and pre-syncope, given the significant overlap and difficulty in differentiating the 2 at the time of evaluation in the ED.

We identified potentially eligible study subjects from chief complaint codes of syncope or dizziness assigned by the ED triage nurse from a pre-populated list. The study excluded patients who were not well-appearing or had a history of cardiac disease, neurologic disease, or other significant co-morbidity, preceding major trauma, or toxic ingestion. These patients mirror the inclusion and exclusion criteria of the EBG. We also excluded children who underwent diagnostic evaluation at outside institutions before transfer. We applied consistent exclusion criteria across the study periods.

### Data Collection and Measures

We performed both chart review and automated abstraction from electronic medical records to collect the following data: demographics, historical information, diagnostic test results [ECG, urine pregnancy test, complete blood count (CBC), serum electrolytes, point of care (POC) glucose, chest radiograph, head computed tomography (CT) scan], cardiology or neurology consultation, IV fluid administration, patient disposition, and length of stay (LOS) associated with each visit. Follow-up data included subsequent visits to the Boston Children’s Hospital ED in the 6 months after the initial ED visit or any outpatient visit to the cardiology clinic or neurology clinic, as well as associated subsequent testing, delayed diagnoses, or death. We collected and managed the data using the Research Electronic Data Capture tools hosted at Boston Children’s Hospital.^18,19^

### Statistical Analysis

We divided the study period into the following 3 segments: (1) the pre-EBG period (the 2 years before the introduction of the EBG, from July 1, 2009 to June 30, 2011); (2) the short-term follow-up period (the year immediately following the introduction of the EBG from July 1, 2011 to June 30, 2012); and (3) the long-term follow-up period (the years following the short-term follow-up period, from July 1, 2012 to December 31, 2017).

With descriptive statistics, we characterized the patients’ demographic features, using frequencies with proportions and medians with interquartile ranges for categorical and continuous variables, respectively. We used chi-square and Kruskal-Wallis rank tests for categorical and continuous variables to assess differences in patient demographics and diagnostic testing rates and other management decisions across periods, respectively.

We evaluated the impact of the EBG on clinical practices in syncope management using a segmented interrupted time series analysis. We chose this method instead of run charts and statistical process control to measure preexisting trends in clinical practices in the pre-EBG period and assess changes in the levels and trends of those practice outcomes across the different periods. The interrupted time series analysis produced 2 tests of interest: the level change and the slope comparison. A significant level change indicates the presence of a one-time shift in the level of the outcome at the beginning of the post-intervention study period, suggesting a significant change in the occurrence of the outcome at the time of its introduction. A significant slope comparison indicates the presence of a change in the trend over time of the outcome between the 2 study periods, suggesting that the intervention accelerated or decelerated change in the rate of the outcome.

First, we estimated a set of regression models with each clinical practice outcome as the dependent variable and time (measured bi-monthly), study period (pre-EBG period versus the short-term and long-term follow-up periods), and the time-by-study period interaction term as the independent variables. We used logistic models for binary outcomes and negative binomial models for the count outcome (LOS). As described above, these models compared the pre-EBG period to the follow-up period rates over time (ie, comparison of slopes) and the pre-EBG period versus follow-up period intercepts (ie, the level-change), while accounting for pre-EBG trends in the outcome. Then, we repeated these analyses while comparing the pre-EBG period to the long-term follow-up period.

Logistic and negative binomial models produce odds ratios (ORs) and incidence rate ratios (IRRs), respectively, as measures of association. An OR or IRR with a 95% confidence interval (CI) that does not include 1 is statistically significant. A CI greater than 1 for a level change assessment indicates an increase in the outcome rate after the intervention. In contrast, a CI less than 1 indicates a decrease in the outcome after the intervention. A CI greater than 1 for a slope comparison indicates an accelerated trend over time in the rate of the outcome after the intervention. A CI of less than 1 indicates a decelerated trend over time in the rate of the outcome after the intervention.

The clinical practice outcomes comprised of testing recommended by the EBG (ECG and urine pregnancy test for post-menarchal female patients) and testing and management not recommended by the EBG (CBC, serum electrolytes, POC glucose, chest radiograph, head CT, cardiology or neurology consultation, IV fluid administration, and admission). We evaluated the LOS of the pediatric ED visit as an outcome, excluding patients with a chief complaint of mental health diagnosis or homelessness across all study periods.

Finally, we calculated 6-month follow-up rates of testing, delayed diagnosis, and death following the introduction of the EBG. Statistical analyses were conducted using Stata SE, version 14 (Stata Statistical Software; StataCorp, College Station, TX). We calculated all tests as 2-tailed and set α level at 0.05.

## RESULTS

### Patient Demographics

In total, 3,426 patients aged 8–22 years during the study period presented with a chief complaint of syncope or dizziness, 2,468 of which had syncope or pre-syncope. After applying the inclusion criteria, the study included 1,294 patients (see Figure 1, Supplemental Digital Content 1, http://links.lww.com/PQ9/A218). No significant differences existed between the study periods in patient demographics, including sex, age, race, ethnicity, and insurance type (Table 1).

**Table 1.** Demographic and Descriptive Characteristics of Children Included in the Study

Demographic Characteristic	Pre-EBG Period (n = 271), July 1, 2009 to June 30, 2011	Short-term Follow-up Period (n = 164), July 1, 2011 to June 30, 2012	Long-term Follow-up Period (n = 859), July 1, 2012 to December 31, 2017	*P*
Sex, female, n (%)	191 (70)	114 (70)	585 (68)	*P* = 0.745
Age in years, median (IQR)	14.7 (12.3–16.8)	14.5 (12.5–16.9)	14.5 (12.4–16.9)	*P* = 0.980
Race, n (%)				*P* = 0.082
White	146 (54)	82 (50)	381 (44)	
Black	42 (16)	25 (15)	137 (16)	
Asian	5 (2)	2 (1)	29 (3)	
Other	78 (29)	55 (34)	312 (36)	
Ethnicity, Latino, n (%)	60 (22)	30 (18)	178 (21)	*P* = 0.631
Primary insurance, n (%)				*P* = 0.205
Private	183 (68)	108 (67)	518 (64)	
Public	87 (32)	54 (33)	313 (36)	

IQR, interquartile range.

### Rates of Evaluations and Interventions for Syncope

Table 2 shows the proportion of patients receiving diagnostic evaluations or interventions for syncope stratified by the study period. Rates of urine pregnancy testing and ECGs significantly increased from the pre-EBG period to the long-term follow-up period (*P* < 0.001 and *P* = 0.001, respectively). Rates of CBC, serum electrolytes, POC glucose, chest radiograph, IV fluid administration, and head CT significantly decreased (all *P* values <0.05).

**Table 2.** Proportion of Patients Receiving Diagnostic Evaluations or Other Management for Syncope in the Pre-EBG Period, Short-Term Follow-up Period, and Long-Term Follow-up Period

Diagnostic Evaluation or Management	Pre-EBG Period (n = 271)	Short-term Follow-up Period (n = 164)	Long-term Follow-up Period (n = 859)
Recommended by the EBG			
ECG	0.92 (0.88–0.95)	0.98 (0.94–0.99)*	0.97 (0.95–0.98)†
Urine pregnancy test‡	0.71 (0.63–0.78)	0.85 (0.76–0.91)	0.85 (0.82–0.88)†
Not recommended by the EBG			
CBC	0.38 (0.33–0.44)	0.21 (0.15–0.28)*	0.11 (0.09–0.13)†
Serum electrolytes	0.34 (0.28–0.40)	0.18 (0.13–0.25)*	0.08 (0.06–0.10)†
Point-of-care glucose	0.31 (0.26–0.37)	0.23 (0.17–0.30)	0.18 (0.15–0.21)†
Chest radiograph	0.15 (0.11–0.20)	0.09 (0.05–0.15)	0.03 (0.02–0.05)†
Head CT	0.03 (0.01–0.06)	0.01 (<0.01–0.04)	0.01 (<0.01–0.02)†
Neurology consult	0.04 (0.02–0.07)	0.04 (0.01–0.08)	0.02 (0.01–0.03)
Cardiology consult	0.07 (0.04–0.11)	0.08 (0.04–0.13)	0.04 (0.02–0.05)
IV fluids	0.32 (0.26–0.37)	0.18 (0.12–0.25)*	0.10 (0.08–0.12)†
Admission	0.03 (0.02–0.06)	0.02 (<0.01–0.06)	0.004 (<0.001–0.01)†

Values in table represent proportion (95% CI).

*P < 0.05 versus short-term follow-up period.

†*P* < 0.05 versus long-term follow-up period.

‡Proportions of post-menarchal females receiving urine pregnancy test.

### Diagnostic Evaluations Recommended by Evidence-based Guideline

**Fig. 2.**
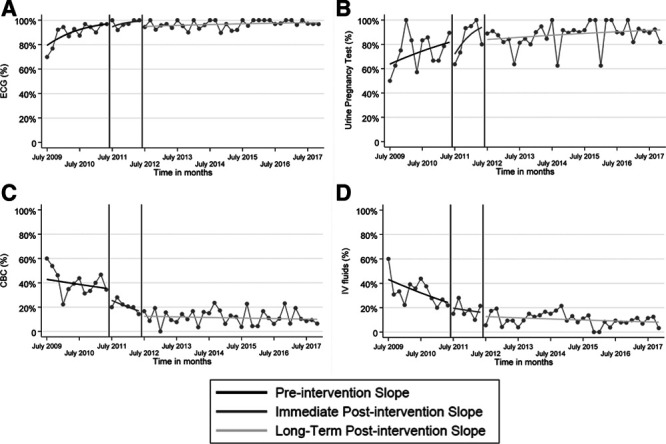
Results of segmented interrupted time series analysis comparing rates of clinical practices in the pre-EBG, short-term follow-up, and long-term follow-up periods. ECGs (A) and urine pregnancy tests in post-menarchal females (B) rose significantly, and CBC (C) and IV fluids (D) declined significantly in the long-term follow-up period.

Table 3 and Figure 2 show the interrupted time series analysis of diagnostic evaluations recommended by the EBG in the pre-EBG period versus the short-term and long-term follow-up periods. Before the EBG, there was a significant trend in rising rates of ECGs in children with syncope (referent slope OR 1.21, 95% CI 1.05–1.39). Still, there were no significant changes in urine pregnancy testing. In the pre-EBG versus the short-term follow-up periods, there were no significant ECGs or urine pregnancy testing changes.

**Table 3.** Segmented Interrupted Times Series Analysis of Diagnostic Testing Recommended by the EBG in the Pre-EBG Period, Short-Term Follow-up Period, and Long-Term Follow-up Period

Outcomes and Corresponding ITS Estimates	Pre-EBG Period Versus Short-term Follow-up Period	Pre-EBG Period Versus Long-term Follow-up Period
ECG	n = 435	n = 1130
Referent slope	**1.21 (1.05–1.39**)	**1.21 (1.05–1.39**)
Level change	3.76 (0.39–36.02)	**5.56 (1.73–17.91**)
Slope comparison	1.20 (0.62–2.34)	**0.85 (0.74–0.99**)
Urine pregnancy test*	n = 222	n = 552
Referent slope	1.09 (0.97–1.22)	1.09 (0.97–1.22)
Level change	1.11 (0.22–5.49)	**3.15 (1.07–9.32**)
Slope comparison	1.32 (0.88–1.98)	0.94 (0.83–1.06)

Values in table represent OR (95% CI).

Bolded ORs are statistically significant.

*Urine pregnancy test for post-menarchal females only.

ITS, interrupted time series analysis.

Rates of ECGs and urine pregnancy testing increased significantly in the long-term follow-up period (level change OR 5.56, 95% CI 1.73–17.91 and 3.15, 95% CI 1.07–9.32, respectively). The trend of obtaining ECGs remained favorable in the long-term follow-up period compared to the pre-EBG period (slope comparison OR 0.85, 95% CI 0.74–0.99).

### Diagnostic Evaluations and Management not Routinely Recommended by Evidence-based Guideline

Table 4 and Figure 2 show the interrupted time series analysis of diagnostic evaluations and management decisions in the pre-EBG period versus the short-term and long-term follow-up periods. Before the EBG, there were no significant trends over time in any outcome. No significant changes occurred in any outcome between the pre-EBG and short-term follow-up periods.

**Table 4.** Segmented Interrupted Time Series Analysis of Diagnostic Testing and Management Not Recommended by the EBG in the Pre-EBG Period, Short-Term Follow-up Period, and Long-Term Follow-up Period

Outcomes and Corresponding ITS Estimates	Pre-EBG Period Versus Short-term Follow-up Period	Pre-EBG Period Versus Long-term Follow-up Period
CBC	n = 435	n = 1,130
Referent slope	0.97 (0.90–1.05)	0.97 (0.90–1.05)
Level change	0.49 (0.17–1.48)	**0.19 (0.09–0.40**)
Slope comparison	0.93 (0.73–1.18)	1.02 (0.94–1.11)
Serum electrolytes	n = 435	n = 1,130
Referent slope	0.94 (0.87–1.02)	0.94 (0.87–1.02)
Level change	0.39 (0.13–1.23)	**0.15 (0.07–0.32**)
Slope comparison	0.97 (0.75–1.25)	1.04 (0.96–1.14)
Point-of-care glucose	n = 435	n = 1,130
Referent slope	0.97 (0.90–1.05)	0.97 (0.90–1.05)
Level change	1.01 (0.34–2.96)	**0.38 (0.18–0.81**)
Slope comparison	0.87 (0.68–1.10)	1.03 (0.95–1.12)
Chest radiograph	n = 435	n = 1,130
Referent slope	0.92 (0.83–1.03)	0.92 (0.83–1.03)
Level change	0.61 (0.14–2.60)	**0.17 (0.06–0.49**)
Slope comparison	0.91 (0.64–1.28)	1.06 (0.94–1.18)
Head CT	n = 435	n = 1,130
Referent slope	0.94 (0.76–1.17)	0.94 (0.76–1.17)
Level change	0.02 (0.00–8.00)	0.13 (0.01–1.38)
Slope comparison	2.00 (0.61–6.56)	1.08 (0.86–1.36)
Neurology consult	n = 435	n = 1,130
Referent slope	0.96 (0.79–1.16)	0.96 (0.79–1.16)
Level change	0.07 (0.00–2.14)	0.70 (0.13–3.77)
Slope comparison	1.78 (0.92–3.46)	1.01 (0.83–1.22)
Cardiology consult	n = 435	n = 1,130
Referent slope	1.02 (0.88–1.18)	1.02 (0.88–1.18)
Level change	0.45 (0.06–3.65)	0.99 (0.25–4.03)
Slope comparison	1.29 (0.86–1.93)	0.95 (0.81–1.10)
IV fluids	n = 433	n = 1,127
Referent slope	0.92 (0.85–1.00)	0.92 (0.85–1.00)
Level change	0.31 (0.10–1.00)	**0.18 (0.08–0.39**)
Slope comparison	1.03 (0.80–1.34)	1.06 (0.98–1.16)
Admission	n = 435	n = 1,130
Referent slope	0.96 (0.78–1.18)	0.96 (0.78–1.18)
Level change	0.02 (0.01–4.33)	0.09 (0.01–1.47)
Slope comparison	1.96 (0.74–5.23)	1.03 (0.81–1.31)

Bolded ORs are statistically significant. ITS, interrupted time series.

In comparing the pre-EBG and the long-term follow-up periods, there were significant level reductions in rates of CBC [level change OR 0.19 (95% CI 0.09–0.40)], serum electrolytes [level change OR 0.15 (95% CI 0.07–0.32)], POC glucose [level change OR 0.38 (95% CI 0.18–0.81)], chest radiograph [level change OR 0.17 (95% CI 0.06–0.49)], and IV fluid administration [level change OR 0.18 (95% CI 0.08–0.39)]. No significant changes occurred in rates of head CT, neurology or cardiology consultation, and hospital admission.

### Length of Stay

The median (interquartile range) LOS was 205 minutes (140–288) in the pre-EBG period, 176 minutes (126–251) in the short-term follow-up period, and 170 minutes (118–234) in the long-term follow-up period. Before the EBG, there was a significant trend in decreasing the LOS for patients with syncope (referent slope IRR 0.98, 95% CI 0.96–0.99). A reduction in LOS in the short-term follow-up period versus the pre-EBG period persisted into the long-term follow-up period [level change IRR 0.68 (95% CI 0.52–0.88) and 0.70 (95% CI 0.59–0.83), respectively]. No significant slope differences in LOS existed among the study periods. In comparison, there were no significant differences in LOS for all patients seen in the ED among the study periods.

### Balancing Measures

Following the introduction of the EBG, 17 patients (1.7%) returned to the Boston Children’s Hospital ED for a similar event within 6 months following their initial visit. Eighty-two patients (8.2%) visited the cardiology clinic, and 31 patients (3.1%) visited the neurology clinic at any time after their initial visit. Twenty-seven patients (2.7%) had echocardiograms, 10 patients (1%) had EEGs, 4 patients (0.4%) had brain MRIs, and 7 patients (0.7%) had laboratory tests. As a result of these encounters following the initial ED visit for syncope, no patients received an alternative serious cardiac or neurologic diagnosis at Boston Children’s Hospital. There were no deaths documented in the Boston Children’s Hospital electronic medical records.

## DISCUSSION

The pediatric ED staff implemented an EBG for syncope in 2011 as a quality improvement intervention. In the following year, relevant testing increased, and low-yield diagnostic studies decreased.^10^ The current research suggests that the EBG continued to influence clinical practices for syncope management for 6 years after its implementation, despite the decrease in resources dedicated to adherence. Interrupted times series analysis identified rising rates of ECGs in children with syncope before introducing the EBG, but no other significant trends in testing existed at that time. Attention to high-value testing amidst rising healthcare costs may have influenced local trends in care involving increasing rates of ECGs before the introduction of the EBG. Follow-up data show that despite the reductions in low-yield diagnostic testing, there were no delayed critical diagnoses.

A robust culture of quality improvement in the Division of Emergency Medicine at Boston Children’s Hospital and the introduction of EBGs for other conditions in the ED during the study period may have influenced the sustained success of the EBG. The proliferation of guidelines and clinical pathways for other conditions may have contributed to the sustained momentum of clinician adherence. Also, the syncope EBG development team included staff from cardiology, neurology, and emergency medicine, representing the best local practices and potentially promoting acceptance by clinicians. Coincident with this EBG, the Department of Cardiology at Boston Children’s Hospital also examined their ambulatory practices in the evaluation of syncope.^20^ The division chief’s annual evaluation of physician performance, including rotating metrics of adherence to a single guideline as a marker of evidence-based practices, likely promotes adherence to the guidelines. Lastly, escalating national discussion on cost containment in healthcare may have impacted clinical practices.

We did not implement intensive efforts to promote a further reduction in rates of tests or interventions that are not routinely recommended by the guideline, including CBC, serum electrolytes, point-of-care-glucose, and IV fluid administration. We do not target complete elimination of this testing but encouraged low rates of these practices in cases lacking concerning features. Furthermore, the resources required to achieve relatively small additional reductions in already low rates of testing may be dedicated to other quality improvement initiatives.

A key lesson from this study is that pediatric EDs can successfully maintain local clinical guidelines to change practices with minimal, focused efforts in the setting of a robust culture of quality improvement. In the context of prior literature supporting the short-term effectiveness of EBGs,^10–13^ our analyses suggest that EBGs are useful in creating sustained changes in clinical practices. Clinicians need simple tools like EBGs to optimize decision-making and support efforts regarding healthcare costs,^21,22^ quality improvement, and implementation of research findings into practice.

The strengths of this study include a large sample size and extended length of the follow-up periods. Limitations of this study include the single-center design at a large, urban, free-standing tertiary care pediatric hospital, which may impair generalizability. Additionally, the follow-up assessment did not include return visits outside of this institution. Lastly, we were unable to control the effects of the implementation of other EBGs on the adherence to this guideline.

Further investigation may shed light on the role of EBGs or clinical pathways for other clinical conditions and different practice settings. Ongoing research should ensure that guidelines do not have unintended consequences regarding outcomes, missed or delayed diagnoses, return visits, or other cost implications.

In conclusion, this study demonstrates that an EBG for syncope management in the pediatric ED influenced long-term changes in clinical practice to promote evidence-based decisions and reduce unnecessary testing without unintended adverse effects. The findings provide strong evidence that local EBGs are a valuable tool to change physician practices in a sustained manner. ED clinicians may utilize EBGs as inexpensive tools to promote safe, cost-efficient, and high-quality management of common diagnoses amid rising health costs in the United States.

## DISCLOSURE

The authors have no financial interest to declare in relation to the content of this article.

## Supplementary Material


