# Supplementary material for: Long-term Effects of an Evidence-based Guideline for Emergency Management of Pediatric Syncope
Source: Pediatr Qual Saf. 2020 Oct 26;5(6):e361. doi: 10.1097/pq9.0000000000000361 (PMC7591128; doi:10.1097/pq9.0000000000000361)
Supplement: Supplementary file 1 [file pqs-5-e361-s001.pdf]

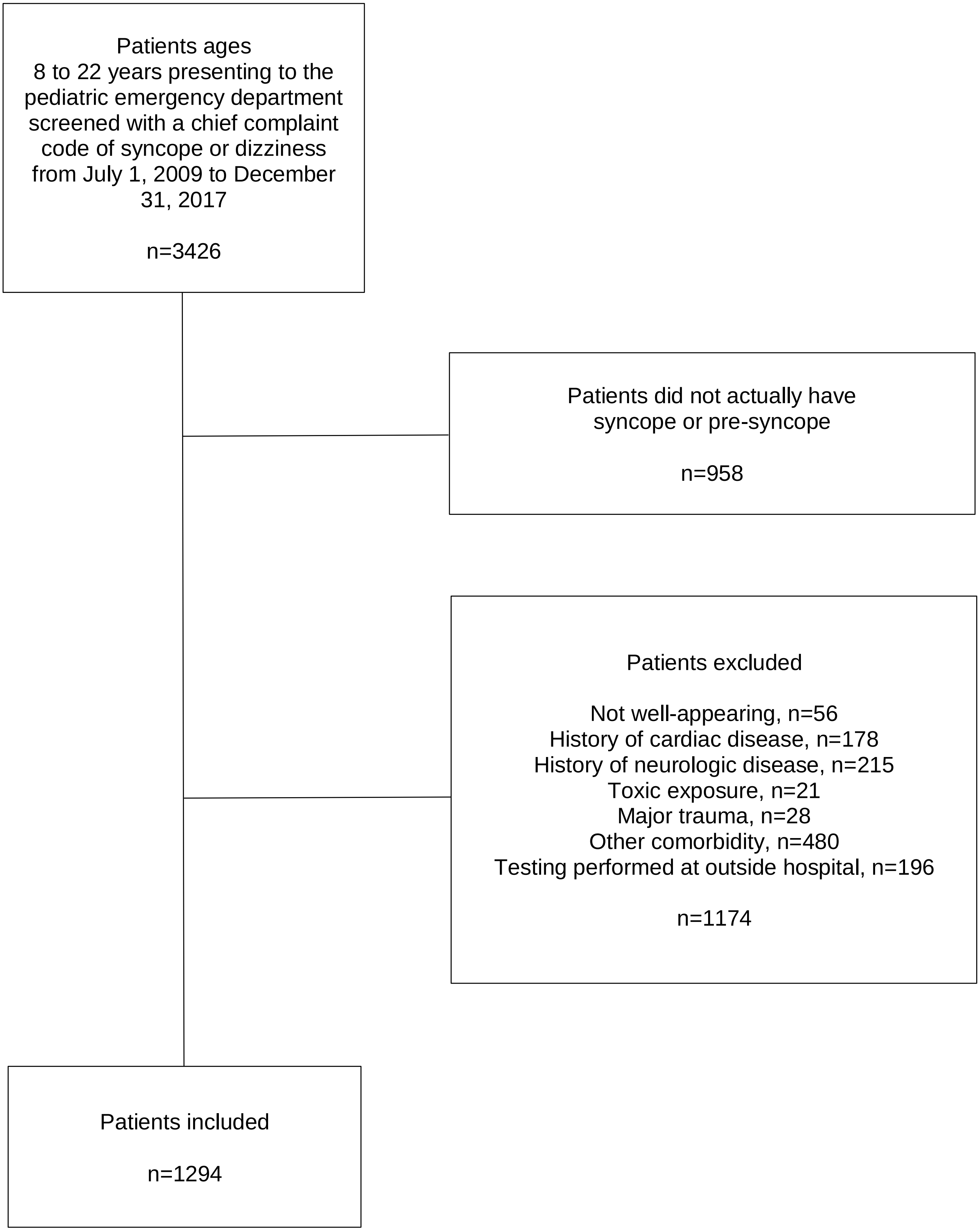

SDC, Figure 1. Flowchart of patients presenting to the pediatric emergency department with syncope before and after the implementation of an evidence based guideline for syncope management in healthy patients aimed to reduce unnecessary testing and promote evidence based clinical practices.
